# Floral Transcriptome Sequencing for SSR Marker Development and Linkage Map Construction in the Tea Plant (*Camellia sinensis*)

**DOI:** 10.1371/journal.pone.0081611

**Published:** 2013-11-26

**Authors:** Li-Qiang Tan, Li-Yuan Wang, Kang Wei, Cheng-Cai Zhang, Li-Yun Wu, Gui-Nian Qi, Hao Cheng, Qiang Zhang, Qing-Mei Cui, Jin-Bo Liang

**Affiliations:** 1 National Center for Tea Improvement, Tea Research Institute of the Chinese Academy of Agricultural Sciences (TRICAAS), Hangzhou, P. R. China; 2 College of Horticulture, Sichuan Agricultural University, Yaan, P. R. China; 3 Tea Research Institute, Enshi Academy of Agricultural Sciences, Enshi, P. R. China; USDA-ARS-SRRC, United States of America

## Abstract

Despite the worldwide consumption and high economic importance of tea, the plant (*Camellia sinensis*) is not well studied in molecular biology. Under the few circumstances in which the plant is studied, *C. sinensis* flowers, which are important for reproduction and cross-breeding, receive less emphasis than investigation of its leaves or roots. Using high-throughput Illumina RNA sequencing, we analyzed a *C. sinensis* floral transcriptome, and 26.9 million clean reads were assembled into 75,531 unigenes averaging 402 bp. Among them, 50,792 (67.2%) unigenes were annotated with a BLAST search against the NCBI Non-Redundant (NR) database and 10,290 (16.67%) were detected that contained one or more simple sequence repeats (SSRs). From these SSR-containing sequences, 2,439 candidate SSR markers were developed and 720 were experimentally tested, validating 431 (59.9%) novel polymorphic SSR markers for *C. sinensis*. Then, a consensus SSR-based linkage map was constructed that covered 1,156.9 cM with 237 SSR markers distributed in 15 linkage groups. Both transcriptome information and the genetic map of *C. sinensis* presented here offer a valuable foundation for molecular biology investigations such as functional gene isolation, quantitative trait loci mapping, and marker-assisted selection breeding in this important species.

## Introduction

Tea continues to be one of the most popular beverages in the world due to its attractive aroma, pleasant taste, and numerous medicinal benefits. In 2011, 3.26 million hectares of tea plant fields were harvested, producing 4.67 million tons of tea (FAO, http://faostat.fao.org/). The tea plant (*Camellia sinensis*) is a woody perennial tree characterized by a large diploid genome (~4 Gb, 2n=30) that is self-incompatible, highly heterozygous, and has a long juvenile period [1]. Such characteristics slow the progress of tea plant breeding: often 22–25 years are required to breed a new tea cultivar through traditional methods [2].

Fortunately, modern biological techniques based on genomic sequencing, DNA molecular markers, and genetic maps will enable us to understand genetic structures of agronomic traits and improve crops rapidly and precisely [3]. However, at this time, the basic genetic resources for *C. sinensis* are relatively scarce. For example, at the beginning of this study (October 2010), only 13,552 nucleotide sequences (most of them expressed sequence tags) of *C. sinensis* were available in GeneBank, and ~200 single sequence repeat (SSR) or microsatellite markers were known [4-8]. In the past two years, more SSR markers for *C. sinensis* have been reported and some of these have been genetically mapped [9-13]. Even so, more SSR markers are required for effective application in genetic mapping and molecular breeding programs for *C. sinensis* and related species. 

Genetic maps are essential tools for implementing quantitative trail loci (QTL) analysis and marker-assisted selection (MAS) breeding. A few genetic maps have been reported recently for *C. sinensis* and these are mainly based on dominant markers such as RAPD or AFLP [14-18]. As quick and inexpensive dominant markers are for map construction, they rarely repeat across plants, so they do not permit easy comparisons of maps at interspecific or intergeneric levels. Thus, such map applications are limited. More recently, Taniguchi et al. constructed a high-density linkage map of *C. sinensis* containing 441 SSRs, 674 of RAPDs and other markers, but this map was limited by the small segregation population that consisted of only 54 F_1_ hybrids of Sayamakaori × Kana-Ck17 [13]. 

Next generation sequencing (NGS) methods, including Illumina and 454, are revolutionary techniques that can produce millions of sequences at a relatively low cost compared with traditional methods [19]. Recently, two research groups published data generated with NGS to sequence the *C. sinensis* transcriptome, which offered a substantial increase in DNA sequence availability for this species. One laboratory sequenced mixed cDNA prepared from seven *C. sinensis* components using Illumina RNA sequencing [1], whereas another laboratory focused on the transcriptome of the leaves using 454 sequencing [20]. Compared to leaf and root research of *C. sinensis* plant, reproductive organs research is underrepresented in public databases. Thus, obtaining gene expression information in these organs will not only expand species genetic resources, but also enable the investigation of molecular mechanisms of flowering and pollination, such as self-incompatibility and various fruit-bearing rates in different cross-parent combinations [21].

Here, we report a deep RNA sequencing study of the *C. sinensis* (cv. Fudingdabaicha) floral transcriptome. We sought to (a) expand the available genomic resources in *C. sinensis*, especially related to its flowers, (b) identify a substantial number of putative SSRs from the obtained transcriptome sequences to develop new SSR markers, (c) and construct a moderately saturated SSR-based genetic linkage map for QTL mapping and MAS breeding in *C. sinensis*. 

## Materials and Methods

### Plant materials, RNA and DNA extraction

For floral transcriptome sequencing, hundreds flowers at the big bud stage of *C. sinensis* (cv. Fudingdabaicha, ten-year-old plant cultivated in the field) were dissected into three parts (petals, pistils and stamens) and immediately snap frozen in liquid nitrogen. Total RNA was extracted from each of these three plant parts using the RNeasy Plant Mini Kit system (Qiagen, Germany) according to the manufacturer's protocol. RNA integrity was confirmed with an Agilent 2100 Bioanalyzer (minimum integrity number = 8). Subsequently, equal amounts of total RNA from the three parts were pooled to prepare a cDNA library. For SSR primer characterization, genomic DNA that served as a PCR template was extracted from eight representative tea cultivars (Fudingdabaicha, Longjin43, Baihaozao, Wuniuzao, Tieguanyin, Huangyan, Changyebaihao and Mingshanbaihao) using the CTAB method.

A segregating F_1_ population consisting of more than 300 individuals was developed at the Tea Research Institute of CAAS at Hangzhou, China using the Longjin43 (♀) and Baihaozao (♂) cultivars as parents. Longjin43 is a runner cultivar for the famous green tea “Xihulongjin”. Baihaozao is high-production cultivar with strong resistance to abiotic stresses, such as drought and cold [22]. Both parents are nationally registered cultivars planted widely in China [2]. Genomic DNA of 188 randomly selected F_1_ individuals were extracted using a DP305 Plant DNA Extraction Kit (Tiangen, Beijing, PR China) following the manufacturer's protocol. 

### cDNA library preparation and Illumina sequencing

The pooled RNA described above was used for cDNA preparation. First, beads with Oligo (dT) were used to isolate poly (A) mRNA. Then a cDNA library was prepared using Illumina's kit according to the manufacturer's recommendations. The *C sinensis* floral cDNA library was sequenced from both the 5’ and 3’ ends using the Illumina HiSeq™ 2000 platform (8 lanes) at the Beijing Genome Institute (BGI, Shenzhen, PR China). The fluorescent images processed for sequence base-calling and quality value calculations were performed using the Illumina data processing pipeline, in which 75 bp paired-end reads were obtained.

### *De novo* transcriptome assembly and analysis of Illumina sequencing results

Before assembly, raw reads were cleaned by removing adaptor sequences, empty reads, reads containing unknown sequences “N” with a rate more than 10%, and low-quality reads containing more than 50% bases with quality score Q-values ≤ 5. *De novo* assembly of clean reads was performed using the SOAPdenovo program [23]. 

Briefly, clean reads were first split into smaller pieces, or ‘k-mers’, for assembly to produce contigs using de Bruijn graphs. The result contigs were then further joined into scaffolds using the paired-end reads. Gap fillings were subsequently carried out to obtain complete scaffolds using the paired-end information to retrieve read pairs that had one read well-aligned on the contigs and another read located in the gap region [24]. Assembled unigene expression was calculated using the reads/kb/million reads (RPKM) method [25].

Files containing the Illumina DNA sequences and quality scores have been submitted to NCBI’s Short Read Archive (accession SRA053025). All assembly unigenes, except those shorter than 200 bp or containing excessive gaps, have been deposited in the Transcriptome Shotgun Assembly (TSA) Sequence Database at NCBI [GeneBank: GAAC01000001–GAAC01052919]. Unigenes not submitted to NCBI are available in Table S1.

### Comparisons with previous transcriptome data of *C. sinensis*

Transcriptome sequences of *C. sinensis* obtained from the Illumina GA IIx platform (accession: HP701085-HP777243) [1] and 454 sequencing platform (accession: KA279444-KA304315) [20] were downloaded from GeneBank. Comparisons of our floral transcriptome with the transcriptome libraries from these 2 resources were performed using a local BLASTn procedure with an E-value threshold of 1e-10.

### Functional annotation and classification

All Illumina-assembled unigenes were annotated by assigning putative gene descriptions, conserved domains, gene ontology (GO) terms, and putative metabolic pathways based on sequence similarity with previously identified genes annotated with those details. For assignments of predicted gene descriptions, the assembled unigenes were compared to the plant protein dataset of NR, Swiss-Prot database, COGs, and the Kyoto Encyclopedia of Genes and Genomes (KEGG) pathways, respectively using a BLASTALL procedure (E-value ≤ 1e-5). 

Functional categorization using GO terms (GO; http://www.geneontology.org) [26] was carried out based on two sets of the best BLASTX hits from the plant protein dataset from the NR database using Blast2GO software [27]. After obtaining GO annotation for unigenes, WEGO software [28] was used to perform GO functional classification of all annotated unigenes. KEGG pathway annotation was performed by sequence comparisons against the KEGG database [29] using the BLASTX algorithm.

At the same time, the orientation of Illumina sequences which failed to be obtained directly from sequencing was derived from BLAST annotations. For other sequences falling beyond the BLAST ESTScan program [30] were used to predict the ‘CDS’ and their orientation.

To determine the number of unique annotations in the four databases: NR, Swissprot, COGs, and KEGG, we filtered the annotation file for redundancy in Nr-ID, Swissprot-ID, COG-ID, and KO-ID, respectively. 

### SSR loci identification and marker development

To understand SSR loci distribution in the *C. sinensis* floral transcriptome library, all unigenes were searched for the presence of SSRs using MISA (http://pgrc.ipk-gatersleben.de/misa/) [31] with the following minimum length criteria (unit size/minimum repeat time): 1/15, 2/6, 3/5, 4/4, 5/4 and 6/3. Compound microsatellites were defined as repeats interrupted by a non-repetitive sequence of a maximum of 100 nucleotides. 

Because longer SSRs tended to be more polymorphic, relatively more strict criteria (unit size/minimum repeat time: 1/20, 2/10, 3/5, 4/5, 5/5 and 6/5) were adopted for the SSRs searching step for marker development. PCR primers were designed for the flanking regions of SSRs using the Primer3.0 program (http://frodo.wi.mit.edu/primer3/) and default parameters [32]. To validate and characterize these primers, a subset of 720 designed primer pairs (CsFM1001~CsFM1720) were synthesized by Invirtrogen (Shanghai, PR China). PCR amplifications using DNA templates extracted from the 8 tea cultivars were performed in 10-µl reaction mixtures, containing 1.0 µl of 10×PCR buffer, 0.2 µl of dNTPs (10 mM), 0.2 µL of each primer (10 µM), 0.5 U of Taq polymerase (TaKaRa, Dalian, PR China) and 20 ng template DNA. Thermocycling conditions were as follows: (1) initial denaturation at 94 °C for 4 min; (2) 35 cycles of 30 sec at 94 °C, 30 sec at 58 °C, and 30 sec at 72 °C; (3) final extension at 72 °C for 10 min. PCR products were resolved on 10% polyacrylamide gels and visualized by silver staining. Each successfully amplified marker was scored for the presence/absence of alleles. The allele number (N_A_), observed heterozygosity (H_O_), and polymorphic information content (PIC) of each marker were calculated by PowerMarker [33].

To investigate whether the new SSR markers amplified the same region or neighboring regions as previously published SSR markers do, DNA sequences used for previous development of SSR markers in the tea plant were retrieved from GeneBank and a local BLAST (E-value ≤ 1e-5) search was performed, comparing these sequences and unigenes for which markers were developed.

### Genotyping in mapping population

A subset mapping population consisting of six F_1_ individuals and two parents was used for screening markers with heterozygosity in Lingjin43 and/or Baihaozao. Sixty SSR markers previously developed by our group were also screened. Of them, 19 SSR markers developed from other transcriptome sequences of *C. sinensis* were published for the first time (prefix “CsSSR”), whereas the other 41 had been reported elsewhere (prefix “A”) [12]. Only informative markers for which one or both parents were heterozygous and different genotypes were observed in the six F_1_ individuals were genotyped in the entire mapping population. PCR amplification and separation of SSR alleles were performed following the methods described above. The scoring of each marker was confirmed to minimize errors.

### Linkage analysis and map construction

JoinMap 4.0 [34] was used for segregation data analysis. Alleles of each marker in the segregation data were called using CP-type population data entry notation in the software. The goodness-of-fit of each marker to the expected segregation ratio was assessed with the Chi-square (χ^2^) test. Markers were grouped with LOD thresholds in the range of 5–10 and a recombination frequency smaller than 0.4. The recombination frequency between markers was converted to map distance using the Kosambi’s mapping function. A consensus map of the two parents was constructed following a “one-step method” as described by Tavassolian and colleagues [35]. Individual maps of the two parents were also constructed using separated data created by the “create maternal and paternal population nodes” function in JoinMap. Distorted markers were included in the map if they did not noticeably affect surrounding marker positions. Linkage groups from the consensus map, Longjin 43, and Baihaozao, were denoted as “LGx”, “Lx” and “Bx”, respectively, where “x” denoted the group series number ordered according to the genetic span of the consensus map. All maps were drawn with MapChart 2.0 [36] and distorted markers were marked with *, **, ***, ****, ***** and ****** for which *P* = 0.05, 0.01, 0.005, 0.001, 0.0005 and 0.0001, respectively. Homologous linkage groups were compared using MapChart 2.0 and illustrated so that each consensus map group was between the parental maps.

Pearson’s correlation coefficient was used to estimate the even distribution of markers over the entire map. The estimated genetic length (*G*_*e*_) was determined by inflating the length of each group of the consensus map by (*m* + 1) / (*m* - 1), where *m* is the marker number on each group [37]. The map genome coverage was calculated by *G*_o_/*G*_*e*_, where *G*_*o*_ was the total length of the consensus map.

## Results

### Sequencing, *de novo* assembly, and sequence analysis

RNA from three parts (petals, pistils and stamens) of *C. sinensis* flowers were extracted, pooled, and sequenced using a Illumina HiSeq™ 2000 platform. A total of 26.9 million clean reads (~2.42 Gbp) in 75 bp was achieved after removing adaptors, abundant sequences, and low-quality reads. An outline of the read assembly is described in Table 1. 

**Table 1:** Statistics for output of RNA-seq and reads assembly of the *C. sinensis* floral transcriptome.

	Sequences	Total Nucleotides(nt)	N50(bp)	Mean length (bp)
Clean reads	26,874,116	2,418,670,440	/	90
Contigs	453,071	62,697,738	113	138
Scaffold	103,918	33,852,032	424	326
Unigene	75,531	30,325,953	480	402

**Figure 1:**
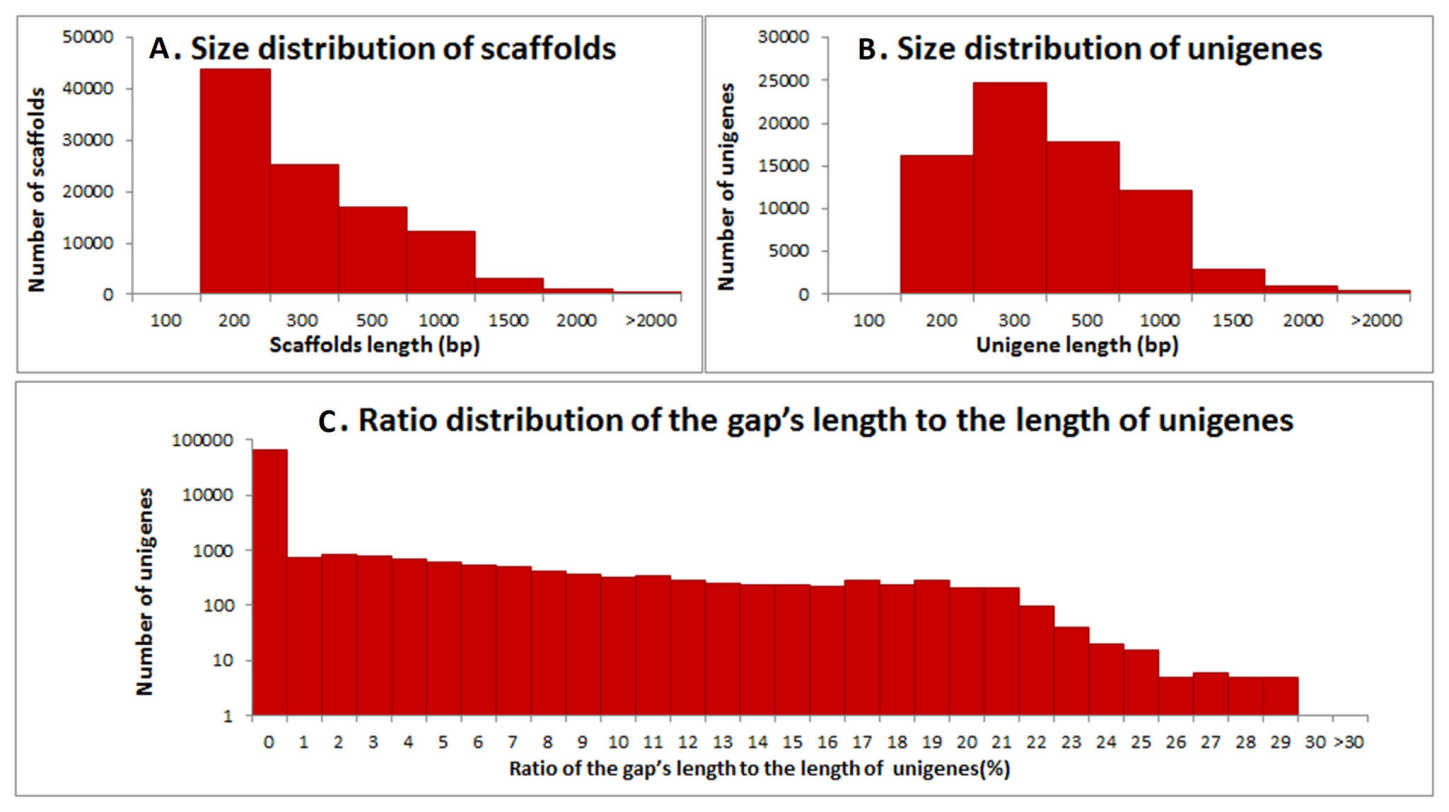
Overview of the *C. sinensis* floral transcriptome assembly.

Using the SOAPdenovo program, all clean reads were assembled into 453,071 contigs (totaling 62.7 Mb), with an average length of 138 bp and an N50 of 113 bp (i.e. 50% of the assembled bases occurred in contigs that were 113 bp or longer). Next, using a contig merging process of paired-end reads, these contigs were joined into 103,918 scaffolds (~33.9 Mb) with an average length of 326 bp and an N50 of 424 bp. Dramatic increases in average lengths were observed in this step. The size distribution of scaffolds is shown in Figure 1A. The proportion of scaffolds with lengths less than 200 bp was reduced to 42.3%, and 17,389 scaffolds (16.0%) with lengths greater than 500 bp were obtained. Finally, by gap filling and contig merging, these scaffolds were joined into 75,531 unigenes, which had sequences with the least Ns (gaps) and no extensions on both ends. Overall, 30.3 Mb bases were successfully assembled into the *C. sinensis* floral transcriptome with a GC content of 0.48. The average sequence length was 402 bp, with an N50 of 480 bp (Figure 1B). Out of the 75,531 unigenes, 17,471 unigenes (21.78%) were ≥ 500 bp. 

To assess the quality of the obtained sequence dataset, we analyzed the ratio of gaps (N) in the assembled unigenes (Figure 1C). In total, 70,475 unigenes (93.3%) had gap lengths less than 5% of the total length. Among them, 66,894 unigenes (88.6%) had no gap. This observation is comparable to previous findings in *C. sinensis* [1] and other non-model species [38].

With BLASTn comparisons we identified that 58,562 (77.5%) and 37,466 (49.6%) unigenes in our floral transcriptome had one or more hits (E value ≤ 1e-10) in *C. sinensis* libraries obtained from the Illumina GA IIx [1] and 454 sequencing platforms [20], respectively. Meanwhile, 51,097 (67.1%) unigenes from the Illumina GA IIx and 23,236 (93.42%) unigenes from 454 sequencing platform were identified as hits in our floral transcriptome library.

### Functional annotation and classification

A total of 50,792 unigenes (67.2%) had BLAST hits to annotated proteins in the NR database, and 31,730 unigenes (42.0%) had BLAST hits to annotated proteins in Swissprot (Table 2). Because different genes can share certain protein domains, multiple DNA sequences will often BLAST-match. After redundancy corrections, the unigene sequences matched 27,223 unique proteins in the NR database and 23,749 unique proteins in Swissprot, indicating that many *C. sinensis* genes are represented. Both the sequence length and expression abundance (represented by RPKM values) of unigenes were positively correlated to the BLAST match rate (data not shown).

**Table 2:** Summary of annotations for the *C. sinensis* floral transcriptome.

	Sequences (n)	Total annotations (n)	Unique annotations (n)	Functional classification
All assembled unigenes	75,531	—	—	—
Gene annotations against NR Gene	50,792	628,678	27,223	—
Gene annotations against Swissprot	31,730	383,703	23,749	—
Gene annotations against KEGG	21,929	21,928	21,929	119 pathways
Gene annotations against COG	13,153	22,505	13,153	25 categories
GO annotations for NR protein hits	22,568	117,338	—	3 main categories 44 sub-categories
All annotated Unigenes	50,792	—	—	—
Unigenes matching all four databases	9,472	—	—	—

**Figure 2:**
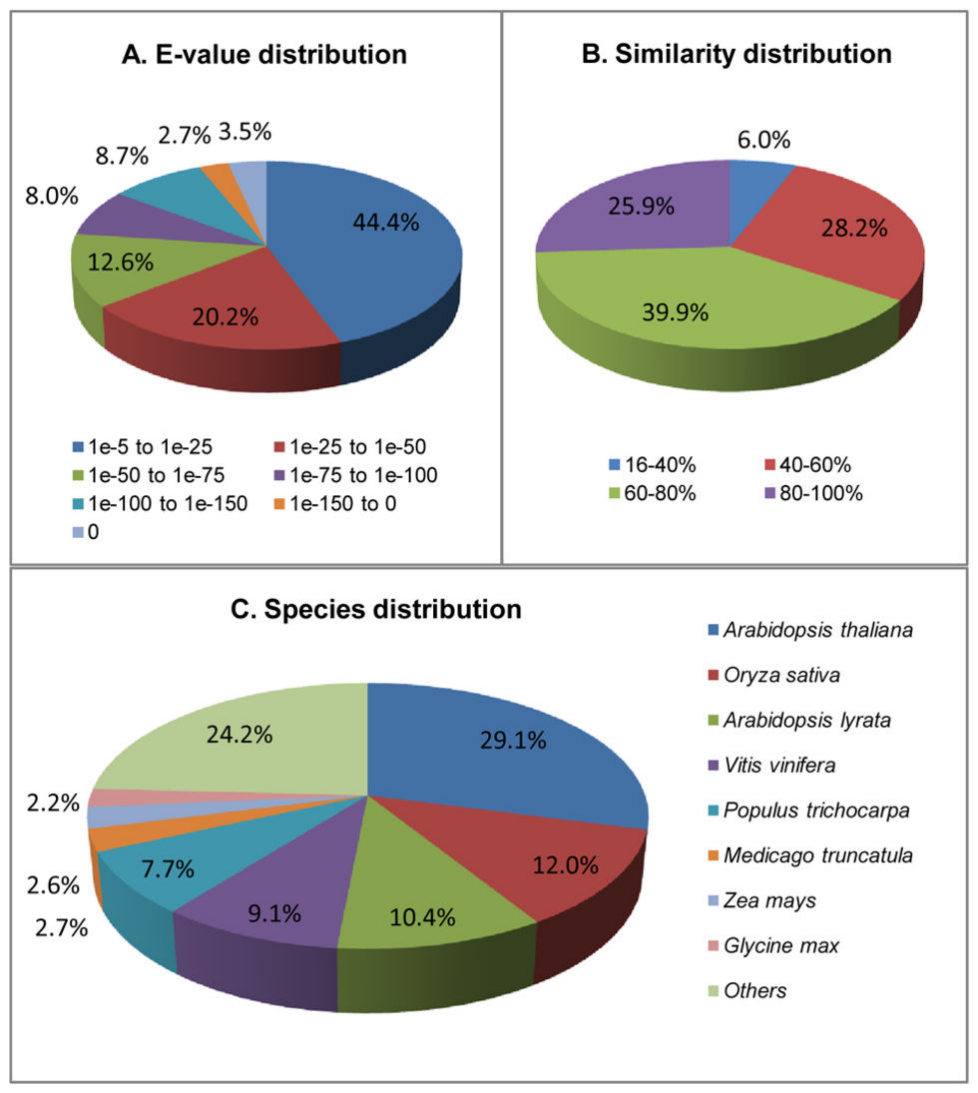
Characteristics of Blast homology search of unigenes against the NR database. (A) E-value distribution of the best matches for unigenes (E-value ≤ 1.0e-5). (B) Similarity distribution of the top BLAST matches for unigenes. (C) Species distribution is shown as the percentage of the total homologous sequences. We blasted total unigene sequences against all plant proteins in NR database and selected the best matches for analysis.

The E-value distribution of the best hits in the NR database showed that 35.4% annotated sequences had high identity with their best hits (smaller than 1e-50), whereas 20.2% ranged from 1e-25 to 1e-50 and another 44.4% ranged from 1e-25 to 1e-5 (Figure 2A). The similarity distribution revealed that 25.9% of the query sequences had greater than 80% similarity, whereas 39.9% of the query sequences varied in similarity from 60–80%, and the other 34.2% varied from 16–60% (Figure 2B). For species distribution, 29.1% distinct unigene sequences were homologous with sequences from *Arabidopsis thaliana*, followed by *Oryza sativa* (12.0%), *Arabidopsis lyrata* (10.4%) (Figure 2C). About 9.1% unigenes were homologous with sequences from *Vitis vinifera*, suggesting a close relationship between *C. sinensis* and *Vitis vinifera.*

Subsequently, unigenes with significant NR database hits were queried against the COG database and 13,153 sequences were aligned to the appropriate COG clusters. Because some unigenes had multiple COG functional annotations, a total of 22,505 functional annotations were obtained and classed into 25 functional categories (see Figure S1 for details). GO classification assigned 117,338 GO terms to 22,568 unigenes that had BLAST hits to annotated proteins in the NR database. These GO terms were classified into three main GO categories and 44 sub-categories by WEGO, indicating a diverse range of functional genes in the floral transcriptome (Figure S2). 

To represent active biological pathways in *C. sinensis* flowers, the 50,792 annotated unigenes were located to the reference canonical pathways in the KEGG database. In total, 21,928 of them were assigned to 119 KEGG pathways. The top three pathways most represented by unique sequences were “biosynthesis of secondary metabolites” (2,637 members), “plant-pathogen interactions” (1,816 members) and “ubiquitin-mediated proteolysis” (572 members), respectively. All annotation information of each unigenes is available in Table S2.

### Characterization of SSR loci

MISA was used to identify SSR loci in the 75,531 unigenes. In total, 12,582 SSRs distributed in 10,290 unigenes (16.67%) were found, roughly one SSR for each 2.41 kb of the transcriptome sequences. There were 1,851 sequences containing two or more SSRs and 1,331 SSR loci were classified as compound forms. With our criteria, di-nucleotide SSRs were most abundant (52.54%), followed by tri-nucleotide SSRs (22.64%) (Table S3). Among di-nucleotide SSRs, the AG/CT motif was most abundant (5,661, 85.60%) followed by AT/TA. Moreover, CG/GC motifs (10, 0.08%) were identified for the first time in *C. sinensis*. The most abundant tri-, tetra-, penta- and hexa-nucleotide SSRs were AAG/CTT (779, 27.35%), AAAT/TTTA (85, 21.63%), AAAAG/CTTTT (60, 20.55%) and AAAAAC/GTTTTT (76, 4.71%), respectively.

### SSR markers development

A total of 2,439 primer pairs were successfully designed from 5,649 flanking regions of long SSRs using Primer 3. Of them, 720 primer pairs were synthesized for verification with a set of 8 tea plant cultivars (including two mapping parents of Longjin43 and Baihaozao). As a result, 431 (59.9%) pairs produced repeatable and polymorphic amplifications and were regarded as new informative markers for *C. sinensis* (Figure S3). The other primer pairs were discarded from further analysis because they (a) were monomorphic in the eight cultivars (97), (b) failed to amplify any product (88), (c) amplified ambiguous products (79) or (d) produced bands larger than 500 bp possibly due to containing long introns (25).

Although only 8 cultivars were used for characterization, a high degree of polymorphism was observed in the 431 new SSR markers. In total, 1,421 alleles were amplified with an average of 3.297 per marker (range 2–7). The average observed heterozygosity (H_O_) is 0.480. The polymorphism information content (PIC) varied from 0–0.815 with an average of 0.464. Searching annotation information (Table S2), 294 out of the 431 unigenes that had been validated as informative marker loci were annotated by BLAST to the Nr database.

We downloaded 630 DNA sequences of *C. sinensis* that had been used for published SSR marker development for which sequence IDs were known [4-6,8,10,12,13]. A local BLAST (E-value ≤ 1e-5) revealed that 18 of 431 unigenes were hits at this threshold, indicating that markers developed from these 18 unigenes were likely amplifying the same regions or neighboring regions as those published SSR markers. Information and characteristics of the 431 novel polymorphic SSR markers are shown in Table S4.

### Genotyping in F1 mapping population

Of the 431 newly developed markers, 262 (60.8%) were heterozygous in Lingjin43 and/or Baihaozao, indicating that they might be mapped through a “pseudo test-cross” approach. We screened the 262 markers with the DNA of two parents and six F_1_ hybrids as PCR templates. Sixty SSR markers of *C. sinensis* developed previously by our group (Table S5) were also screened using the same method. In total, 244 SSR markers (213 developed here and 31 previously developed) were informative in the subset population and were genotyped in the whole mapping population (Figure S3). The remaining 78 markers were excluded due having no detectable polymorphism between parents or because the alleles did not segregate in the 6 F_1_ hybrids. 

According to JoinMap 4.0 CP population data entry notations, 244 genotyped markers could be classified into five categories: 25 were fully informative with four alleles (ab×cd); 60 were fully informative with three alleles (ef×eg); 23 were segregated in both parents with two of the same alleles (hk×hk); 76 were maternally informative (lm×ll); and 60 were paternally informative (nn×np). 

Of the 188 F_1_ hybrids, 15 were identified as fake crosses between Longjin43 and Baihaozao due to the presence of unexpected alleles, so they were excluded from the linkage analysis. Data from 3 other hybrids were also excluded due to a high degree of absence, likely due to poor DNA quality. Eventually, segregation data from 170 F_1_ hybrids were used for the linkage analysis. 

Segregation distortion (*P* < 0.05) was observed for 44 markers (18.03%) in the combined data. Maternal parent Longjin43 had a relatively high skewed segregation proportion (21.1%) compared with the paternal parent Baihaozao (8.3%) as observed in the individual segregation data created by JoinMap.

### Linkage map construction

**Figure 3:**
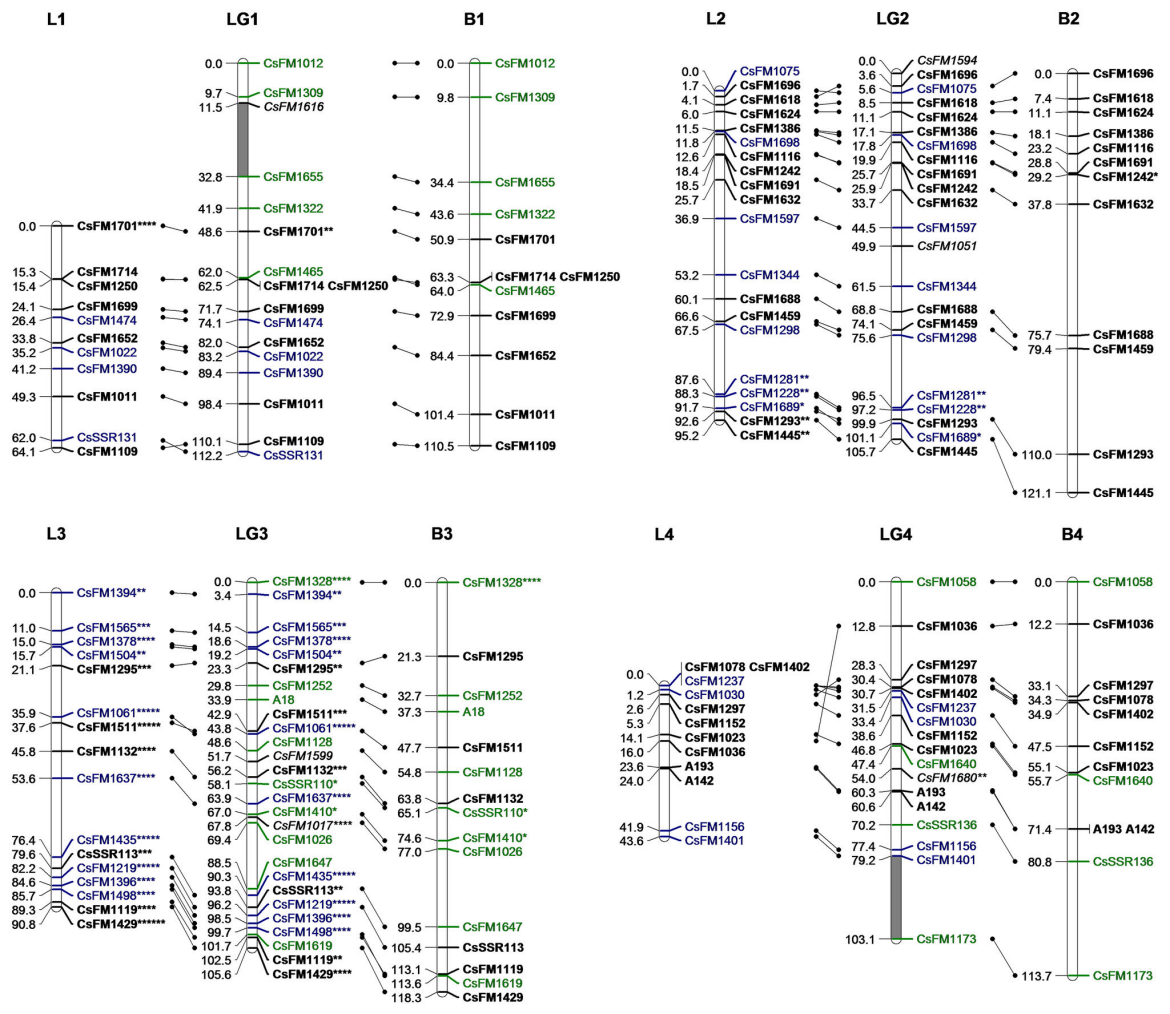
Linkage map based on SSR markers for *C*. *sinensis* (LG1-4). The map in the middle (LG1–LG15) is the consensus map constructed from the combined dataset. The map on the left (L1–L15) and right (B1–B15) are the maternal (Longjin43) and parental (Baihaozao) maps, respectively. Homology between these is depicted by lines with dots on both ends. Markers to describe both parents are bold (ab×cd or ef×eg), markers for Longjin43 only are blue (lm×ll), markers for Baihaozao only are green (nn×np). Italic markers are hk×hk segregation types. Grey bars in the consensus map indicate gaps longer than 20 cM. Distorted markers are indicated with *, **, ***, ****, ***** and ******, where *P* = 0.05, 0.01, 0.005, 0.001, 0.0005 and 0.0001, respectively.

**Figure 4:**
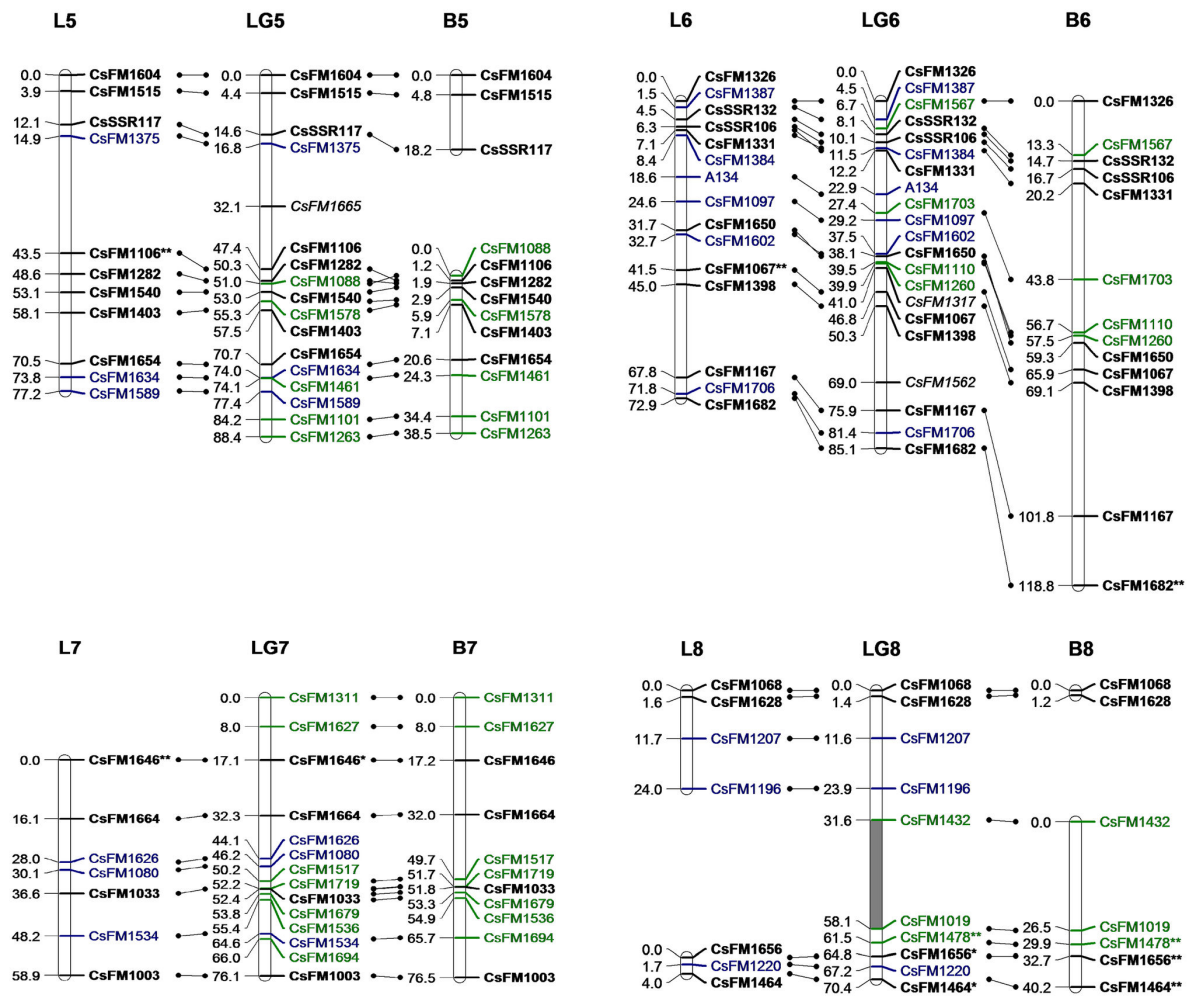
Linkage map based on SSR markers for *C*. *sinensis* (LG5-8). See legend of Figure 3.

**Figure 5:**
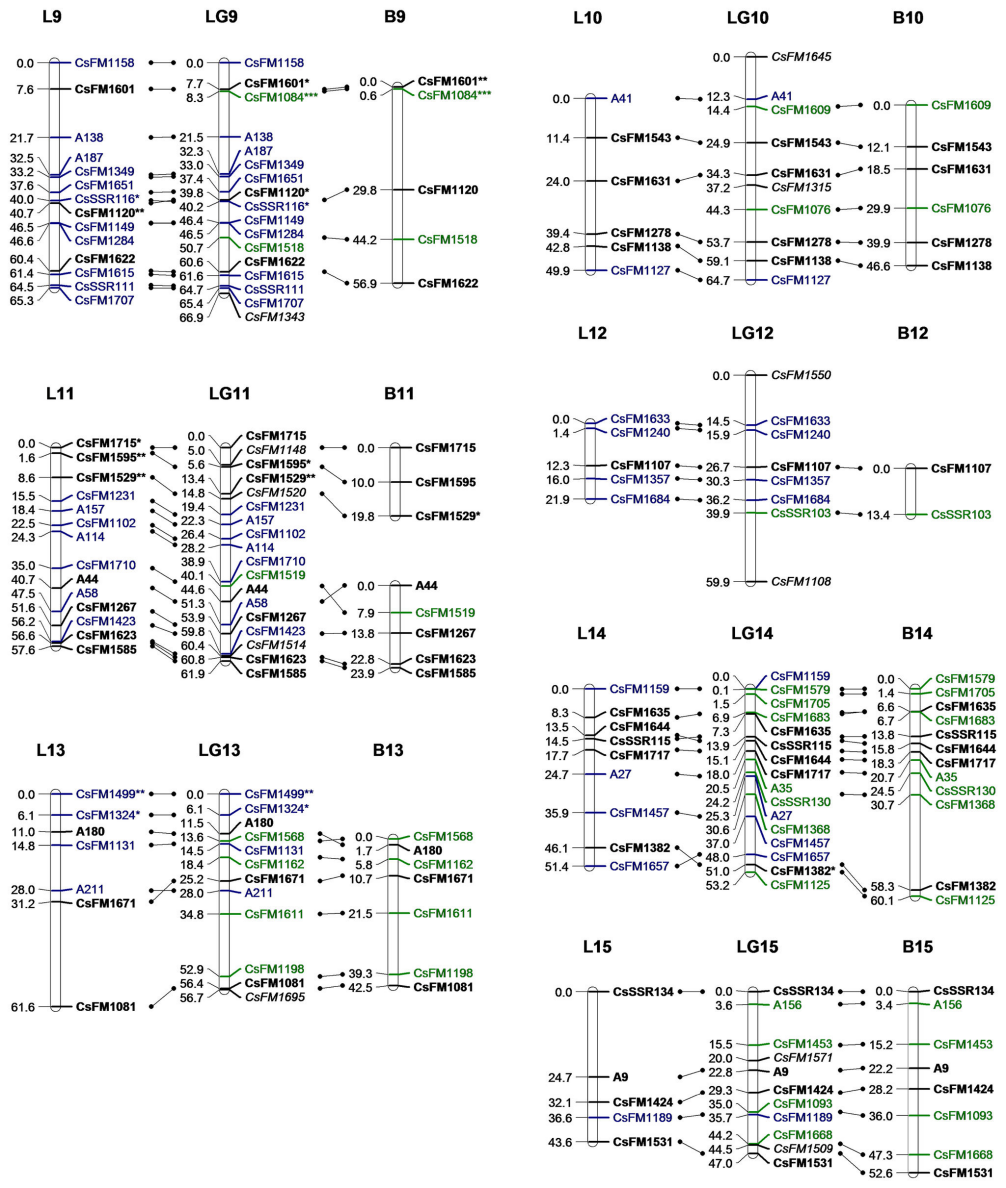
Linkage map based on SSR markers for *C*. *sinensis* (LG9-15). See legend of Figure 3.

A consensus map and two individual maps of the two parents were constructed by JoinMap 4.0. They are illustrated using three lines that depict each linkage group of the consensus map between the homologous components from two individual maps (Figure 3-5). 

The consensus map consisted of 237 SSR markers distributed in 15 linkage groups (LG1–15, numbered sequentially according to their genetic spans, Table 3). Six distorted markers were excluded because their existence changed the order of nearby markers or significantly increased the map distance. The remaining single normally segregated marker was unmapped due to weak linkages to other markers. The number of linkage groups was consistent with the haploid chromosome number in *C. sinensis* (n=15). The total map distance was 1,156.9 cM, with an average marker interval of 5.2 cM. Three gaps greater than 20 cM were found on LG1, LG4 and LG8.

**Table 3:** Characteristics of each linkage group of the consensus map.

Linkage groups	Map length (cM)	No. of markers	Average distance (cM)	No. of distorted markers
LG1	112.2	17	7.01	1
LG2	105.7	22	5.03	3
LG3	105.6	27	4.06	20
LG4	103.1	17	6.44	1
LG5	88.4	17	5.53	0
LG6	85.1	21	4.26	0
LG7	76.1	14	5.85	1
LG8	70.4	10	7.82	3
LG9	66.9	17	4.18	4
LG10	64.7	10	7.19	0
LG11	61.9	18	3.64	2
LG12	59.9	8	8.56	0
LG13	56.7	12	5.15	2
LG14	53.2	16	3.55	1
LG15	47.0	11	4.70	0
Total/average	1156.9	237	5.21	38

Of the 237 mapped SSR markers in the consensus map, thirty-eight were distorted at varied degrees and 20 were clustered at LG3, accounting for 74.1% of the marker numbers in this group. The remaining 18 distorted markers were interspersed within other 9 linkage groups, often in clusters of two or three distorted markers. 

The individual map of Longjin43 contained 16 groups with 159 SSR markers and the total genetic span was 882 cM. The map of Baihaozao contained 18 groups with 142 SSR markers that covered 1,072cM. All groups of the two individual maps were aligned to the consensus map. Due to the lack of informative markers in the interspaces, L8, B4, B8 and B11 were divided into two groups (Figure 3-5). Good synteny of markers between the consensus map and the individual maps was found, despite a few minor inversions in marker orders. Pearson’s correlation coefficient between the genetic length and the marker number in each group of the consensus map was 0.67, indicating that the markers were approximately evenly distributed in the map. The expected genetic length of *C. sinensis* was estimated to be 1,322.9 cM by inflating each group of the consensus map by (m+1) / (m-1) [37]. Therefore, the consensus map covered about 87.4% of the *C. sinensis* genome.

## Discussion

### Transcriptome assembly, functional annotation and classification

Illumina RNA sequencing technology is an extremely high-throughput and cost-effective way to obtain large amounts of transcriptome data and it is a proven tool for transcriptome sequencing of non-model organisms [1,19]. In the present study, a total of 26.9 million clean reads with 75 bp were achieved using Illumina RNA sequencing and 75,531 unigenes from the *C. sinensis* floral transcriptome with an average length of 402 bp were assembled. These unigenes were longer than those identified in previous *C. sinensis* Illumina sequencing [1] in which the average unigene length was 355 bp. However these unigenes were shorter than those identified by Wu and colleagues (733 bp) due to the different sequencing techniques applied [20].

BLAST annotation of unigenes to 27,223 unique proteins suggested our data extensively covered the *C. sinensis* floral transcriptome. Overall, 67.7% of the assembled unigenes had BLAST hits in at least one of five public databases and 9,472 unigenes had hits and defined functional annotations in all five public databases (Table 2). In total, 31,730 unigenes were annotated by blasting the best hits to 23,749 unique proteins in Swissprot. Meanwhile, 21,928 unigenes were assigned to 119 KEGG pathways. Both gene function annotations and the representation of active biological pathways indicated diverse functional genes in the floral transcriptome data. 

These unigene sequences and their annotations provide valuable genetic resources for investigating specific pathways or processes and exploiting new functional plant genes such as the use of several unigenes correlated to gametophytic self-incompatibility (Of note, these unigenes were not found in previously published nucleotide sequences of *C. sinensis*), we cloned full-length cDNA of the S-RNase gene of *C. sinensis* which is believed to have a key role in self-incompatibility [39] (data forthcoming).

### Abundance and distribution of SSR loci

In the study, 12,582 SSRs were identified from 75,531 *C. sinensis* floral unigenes, substantially increasing the availability of a SSR resource for marker development in this economically important species. Approximately 16.67% unigenes contained SSRs, a value higher than previous reports [10,11]. 

Here, we identified all four types of di-nucleotide motifs (considered the base pairing) in the *C. sinensis* floral transcriptome. AG/TC was the most frequent, accounting for 85.6% of the di-nucleotide SSRs. This proportion was consistent with previous reports in *C. sinensis* [9,10], and was comparable with data for Arabidopsis (83%) and apple (88%) [40,41], but much higher than other plants (74% in strawberry, 41% in citrus or 38% to 59% in cereal species such as barley, maize, rice, sorghum and wheat) [42-44]. CG/GC SSRs were reported to be completely absent in the ESTs of *C. sinensis* [5,7-11], however, ten CG/GC SSRs with repeats of 6–8 were detected in our study, accounting for 0.08% of the total di-nucleotide SSR. Low frequencies of CG/GC repeats were also observed in the ESTs of other plants [41,44]. The tendency of CpG sequences to be methylated, an event which might inhibit transcription, was suggested to explain the strong bias against CG/GC repeats in ESTs or transcriptome sequences [41,45]. For tri-nucleotide SSRs, the AAG/CTT repeats were the most abundant motifs identified in our work and they were also the most abundant motifs in *Arabidopsis* and in apple [41]. In our study, di-nucleotide SSRs significantly outnumbered tri-nucleotide SSRs, a finding that was consistent with previous studies of the tea plant [10,20], although the SSRs proportions are greatly affected by the searching criteria used.

### SSR marker development

SSR markers are robust tools for genetic mapping and molecular breeding in crops. SSR marker development feasibility for non-model organisms has been enhanced with NGS technology which can generate large amounts of sequence data [19]. Here, we report that 2,439 SSR primer pairs were successfully designed and 720 of them were tested using eight representative tea cultivars. We verified that ~59.9% could be used as polymorphic markers. Our success rate was comparable to data from previous reports in which SSR markers were developed from ESTs of *C. sinensis* [7-10]. 

The new SSR markers reported here can be used for genetic mapping in our population (see below) and others, including related species of *C. sinensis* due to the high transferability of gene-based and multi-allelic traits [5,7-10]. Thus, these markers can facilitate the QTLs analysis, comparative mapping, and MAS breeding in the tea plant. 

### SSR-based Linkage map

Due to the shortage of SSR markers, most genetic maps of *C. sinensis* previously reported were based on dominant markers, such as RAPDs and AFLPs [13-18]. We isolated thousands of candidate SSR markers using NGS techniques and constructed a moderately saturated SSR-based genetic map of *C. sinensis*. 

In the consensus map, 237 SSR markers were mapped onto 15 linkage groups, presumably corresponding to the 15 chromosome pairs of *C. sinensis*. The total genetic distance of the consensus map was 1,156.9 cM. The estimated genome coverage was 87.4% and the average interval between markers was 5.2 cM, which is sufficient for a genome-wide search for QTL of important traits. To cover the entire genome and fill the large gaps that were observed in LG1, LG4 and LG8, more SSR or SNP can be added to this map in future investigations.

Although 18 individuals were discarded because of false-cross or poor genotype data, the mapping population used here included 170 F_1_ individuals. To our knowledge, this is the largest map population reported in *C. sinensis*. Furthermore, this population can be expanded to more than 300 individuals and at this time, they have been planted under the same conditions and traits are being recorded. The genetic map reported is useful for positioning QTLs involved in important agronomic traits, such as disease-resistance, tea yield and quality. Also, the map offers promise for fine QTLs analysis, MAS breeding, and positional cloning after integration of more SSRs or SNPs.

### Genetic map length

The total map length in this study (1,156.9 cM) is comparable to the distance of the SSR-based core map reported by Taniguchi et al. (1,218cM) [13], but shorter than other reports (varied from 1,349.7–4,482.9cM) that were mainly based on dominant markers [14,15,18]. The genetic map length of one species could be affected by several factors such as map coverage, the mapping population, errors in genotyping and the choice of mapping functions. It is also common that the linkage group length varied among different maps but the marker order was generally similar [46]. This occurred with LG2, LG4 and LG6 in our work presented here. Nevertheless, the length difference of the *C. sinensis* genetic map from our and Taniguchi’s studies [13] and the work by Hu et al [18] is large, and warrants further study. 

### Segregation distortion

Segregation distortion is common in mapping studies of trees and many distorted markers may be associated with alleles affecting viability [47]. In previous *C. sinensis* mapping efforts, distorted loci were normally discarded from linkage analysis [14,16,17], but this is unnecessary because: (a) most had little influence on map order [46-48] and, (b) they may have been linked to genes or traits of interest [49]. In fact, skewed markers were utilized in many genetic mapping studies of trees [35,46-50]. 

In present study, we found 44 loci out of the 244 genotyped markers (18.03%) deviating significantly (*P* ≤ 0.05) from the expected Mendelian segregation ratio. They were not discarded from the linkage analysis unless their existence disturbed flanking marker order or markedly increased the map distance (which occurred for a few markers that distorted at *P* ≤ 0.0001). As a result, 38 distorted markers were mapped on the consensus map, and more than half of these were clustered into one group (LG3). Furthermore, 13 out of the 14 mapped severely distorted markers (marked with three or more asterisks, *P* ≤ 0.005) occurred on LG3. Comparing LG3 to the homologous individual groups of both parents, we confirmed that most of the distorted markers in LG3 were attributed to the female parent (Longjin43). Despite the high rate of distorted markers, LG3 markers appeared to be mapped correctly due to the high synteny of common markers to the B3 group where most markers were normally segregated. This finding indicates that one or more deleterious genes may be within the LG3 of Longjin43 but not in the same region of Baihaozao. So investigating a potentially deleterious gene(s) may give insight to the low fruit-bearing rates of *C. sinensis*.

## Conclusions

In this study, we profiled the floral transcriptome using NGS technology, characterized the SSR loci, and isolated thousands of candidate SSR markers in *C. sinensis*. We also developed 431 novel polymorphic SSR markers and constructed a moderately saturated linkage map for this species. This wealth of DNA sequence and annotation information will provide a valuable resource for functional gene isolation and molecular marker development. The SSR-based linkage map should provide a solid foundation for tea plant QTLs mapping and MAS breeding. Also, the data offer a framework linkage map which can be quickly saturated with SNPs from future studies.

## Supporting Information

Table S1 **Unigenes not submitted to GeneBank.**
(XLSX)

Table S2 **Annotation information of the 75,531 unigenes obtained from *C. sinensis* floral transcriptome.**
(XLSX)

Table S3 **Distribution of different repeat motif SSRs identified in *C. sinensis* floral transcriptome.**
(DOCX)

Table S4 **Characteristics of the 431 SSR markers for *C. sinensis* in present study.**
(XLSX)

Table S5 **The 60 other SSR markers used in present study.**
(XLSX)

Figure S1 **COG function classification for unigenes from *C. sinensis* floral transcriptome.**
(TIF)

Figure S2 **Histogram presentation of Gene Ontology classification of *C. sinensis* floral transcriptome.**
(TIF)

Figure S3 **Some gel images of the experiments.**
(PDF)
