# Supplementary material for: Floral Transcriptome Sequencing for SSR Marker Development and Linkage Map Construction in the Tea Plant (Camellia sinensis)
Source: PLoS One. 2013 Nov 26;8(11):e81611. doi: 10.1371/journal.pone.0081611 (PMC3841144; doi:10.1371/journal.pone.0081611)
Supplement: Table S3 — Distribution of different repeat motif SSRs identified in C. sinensis floral transcriptome. (DOCX) [file pone.0081611.s003.docx]

**Table S3 Distribution of different repeat motif SSRs identified in *C. sinensis* floral transcriptome.**

| **Repeat motif** | **Repeat number** | | | | | | | | | | | **Total** | **%** |
| --- | --- | --- | --- | --- | --- | --- | --- | --- | --- | --- | --- | --- | --- |
|  | 3 | 4 | 5 | 6 | 7 | 8 | 9 | 10 | 11−15 | 16−20 | >20 |  |  |
| Mon-nucleotide |  |  |  |  |  |  |  |  | 159 | 407 | 259 | **825** | **6.56%** |
| Di-nucleotide |  |  |  | 1,439 | 1,120 | 831 | 718 | 716 | 1,702 | 70 | 15 | **6,611** | **52.54%** |
| Tri-nucleotide |  |  | 1,414 | 656 | 331 | 301 | 101 | 17 | 27 | 1 |  | **2,848** | **22.64%** |
| Tetra-nucleotide |  | 263 | 84 | 36 | 10 |  |  |  |  |  |  | **393** | **3.12%** |
| Penta-nucleotide |  | 181 | 101 | 10 |  |  |  |  |  |  |  | **292** | **2.32%** |
| Hexa-nucleotide | 1,088 | 457 | 47 | 15 | 5 | 1 |  |  |  |  |  | **1,613** | **12.82%** |
| **Total** | **1,088** | **901** | **1,646** | **2,156** | **1,466** | **1,133** | **819** | **733** | **1,888** | **478** | **274** | **12,582** | **100.00%** |
