# Supplementary figures and images for: Floral Transcriptome Sequencing for SSR Marker Development and Linkage Map Construction in the Tea Plant (Camellia sinensis)
Source: PLoS One. 2013 Nov 26;8(11):e81611. doi: 10.1371/journal.pone.0081611 (PMC3841144; doi:10.1371/journal.pone.0081611)

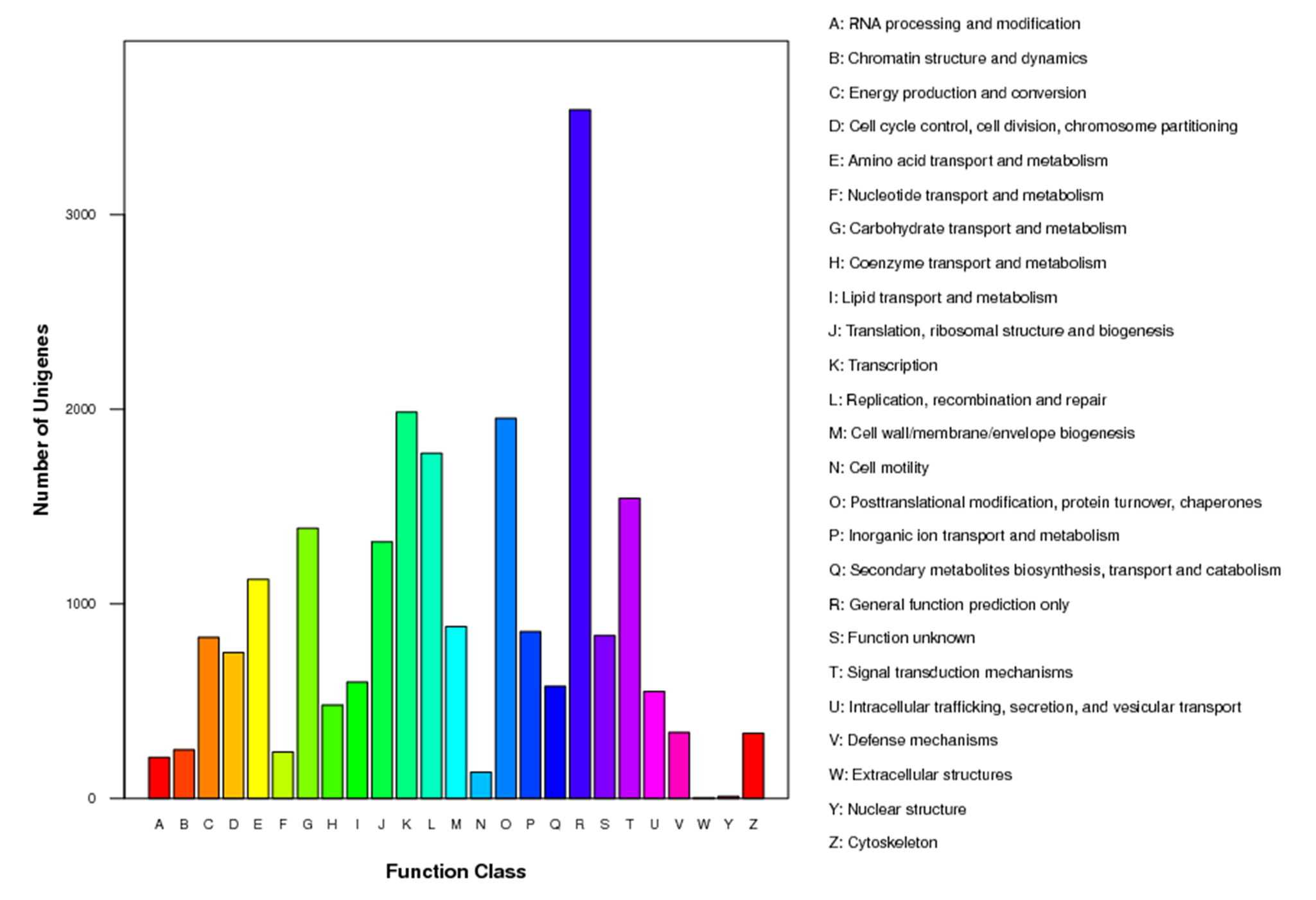

Supplement: Figure S1 — COG function classification for unigenes from C. sinensis floral transcriptome. (TIF) [file pone.0081611.s006.tif]

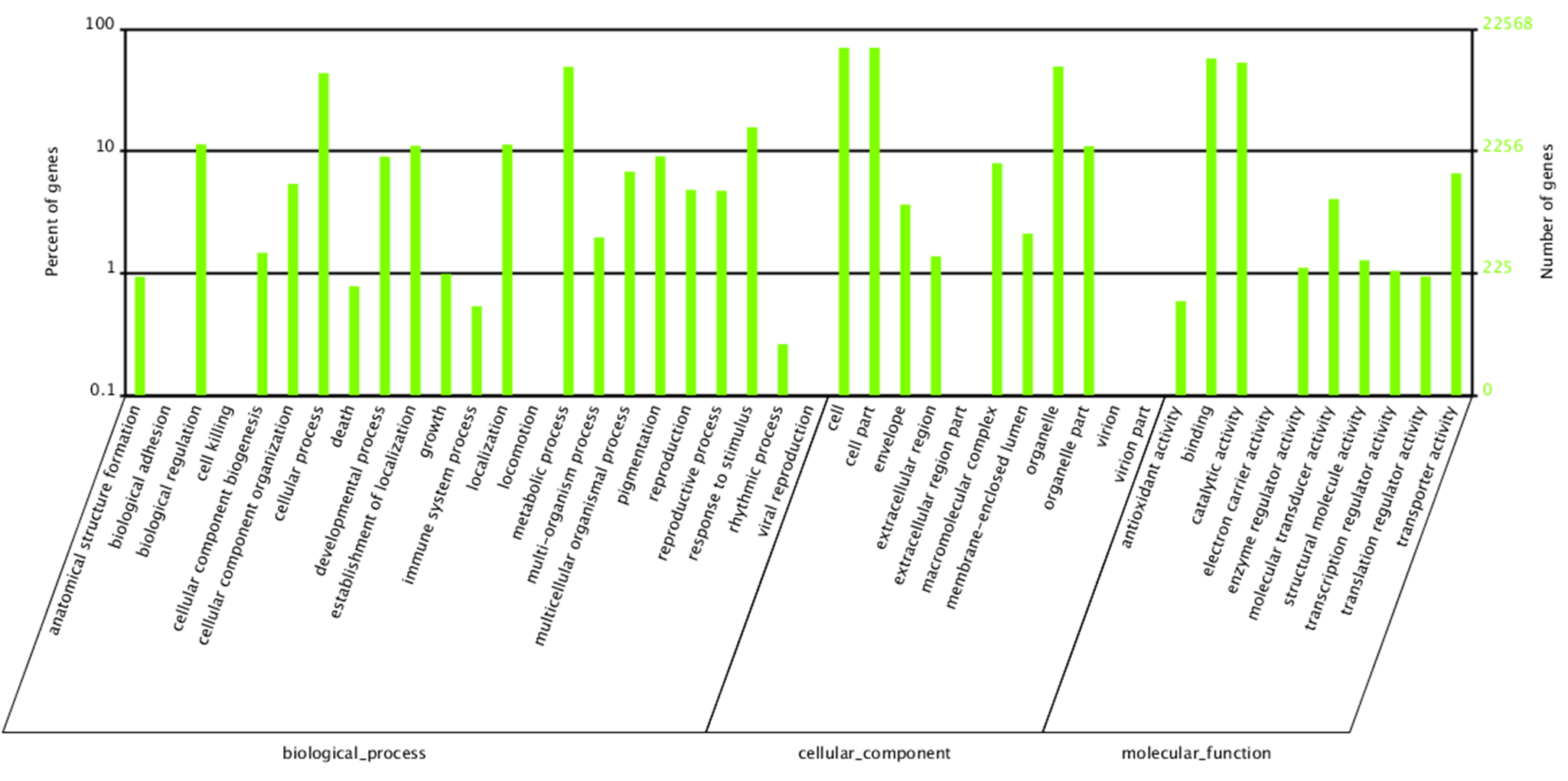

Supplement: Figure S2 — Histogram presentation of Gene Ontology classification of C. sinensis floral transcriptome. (TIF) [file pone.0081611.s007.tif]
