# Supplementary material for: Floral Transcriptome Sequencing for SSR Marker Development and Linkage Map Construction in the Tea Plant (Camellia sinensis)
Source: PLoS One. 2013 Nov 26;8(11):e81611. doi: 10.1371/journal.pone.0081611 (PMC3841144; doi:10.1371/journal.pone.0081611)

Supporting Image 1. Genomic DNA extracted from F_1 hybrids using a DP305 plant DNA extraction kit (Tiangen, Beijing, PR China).

Genomic DNA was resolved on 1% agarose gel and visualized by ethidium bromide staining.

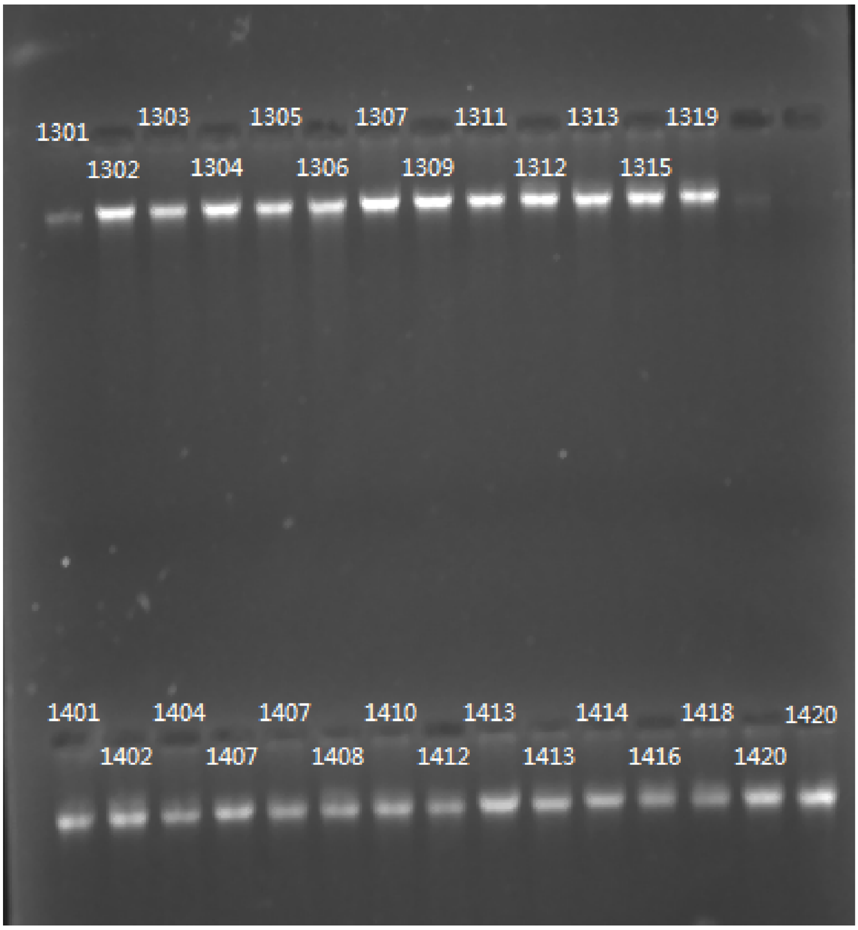

Supporting Image 2. Validation and characterization of SSR markers with 8 tea plant cultivars.

PCR products were resolved on 10% polyacrylamide gel and visualized by silver staining. Lanes 1–8: Changyebaihao, Fudingdabaicha, Baihaozao, Longjin43, Wuniuzao, Huangyan, Tieguanyin and Mingshanbaihao; M: DNA ladder

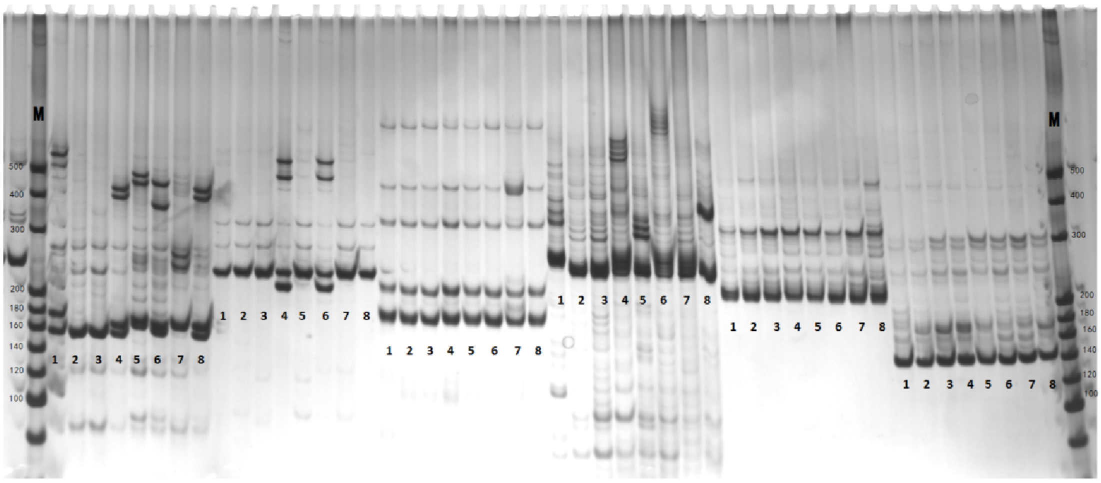

Supporting Image 3. Genotyping in the mapping population

(Marker: CsFM1459; targeted size: 253bp; segregation type: ef × eg)

PCR products were resolved on 10% polyacrylamide gels and visualized by silver staining. ♀ (lanes 47 and 143): Longjin43; ♂ (lanes 48 and 144): Baihaozao; lanes 1–46, 49–142 and 145–192: F_1 hybrids; M: DNA ladders.

P1-1

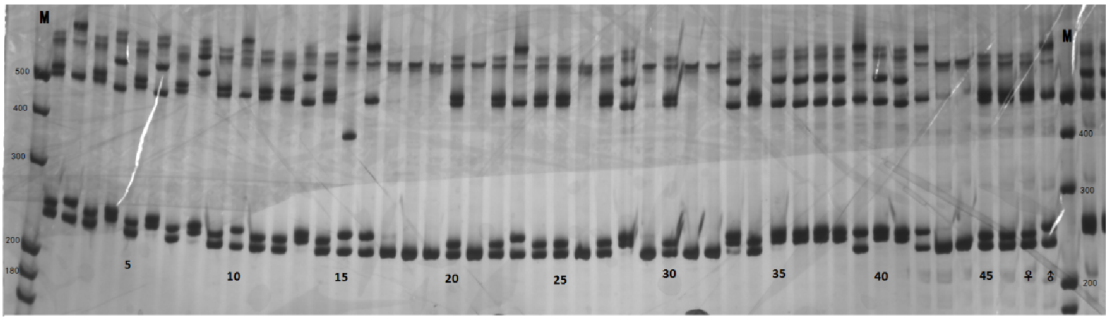

P1-2

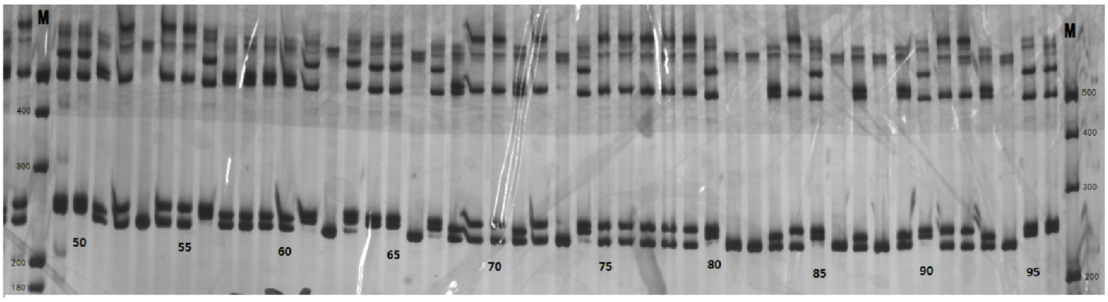

P2-1

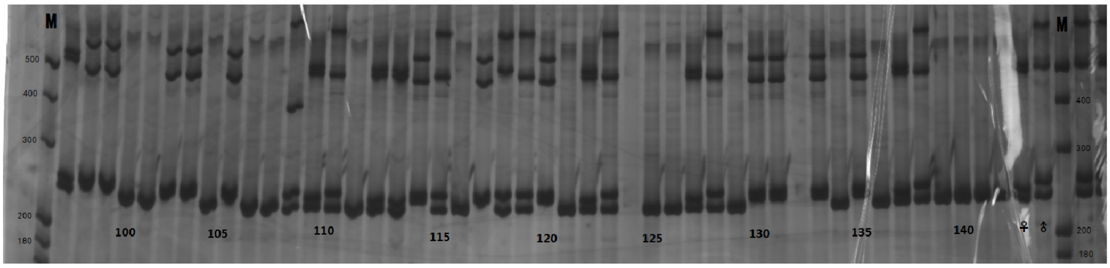

P2-2

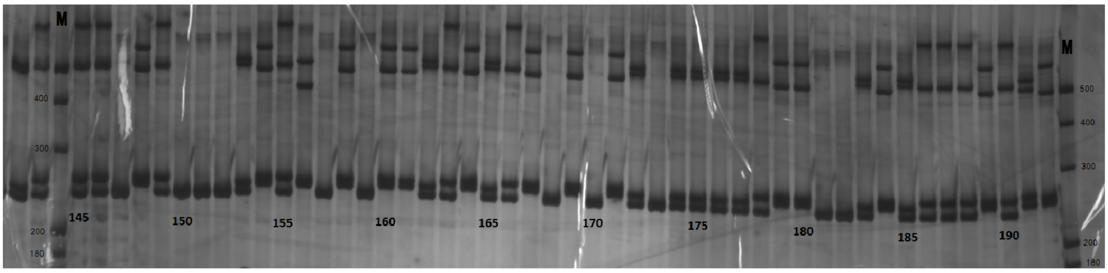

Supplement: Figure S3 — Some gel images of the experiments. (PDF) [file pone.0081611.s008.pdf]
